# Tartronate Semialdehyde Reductase Defines a Novel Rate-Limiting Step in Assimilation and Bioconversion of Glycerol in *Ustilago maydis*

**DOI:** 10.1371/journal.pone.0016438

**Published:** 2011-01-31

**Authors:** Yanbin Liu, Chong Mei John Koh, Longhua Sun, Lianghui Ji

**Affiliations:** Biomaterials and Biocatalysts Group, Temasek Life Sciences Laboratory, 1 Research Link, National University of Singapore, Singapore, Singapore; Institute of Developmental Biology and Cancer Research, France

## Abstract

**Background:**

Glycerol is a by-product of biodiesel production. Currently, it has limited applications with low bioconversion efficiency to most metabolites reported. This is partly attributed to the poor knowledge on the glycerol metabolic pathway in bacteria and fungi.

**Methodology/Principal Findings:**

We have established a fast screening method for identification of genes that improve glycerol utilization in *Ustilago maydis.* This was done by comparing the growth rates of T-DNA tagged mutant colonies on solid medium using glycerol as the sole carbon source. We present a detailed characterization of one of the mutants, GUM1, which contains a T-DNA element inserted into the promoter region of UM02592 locus (MIPS *Ustilago maydis* database, MUMDB), leading to enhanced and constitutive expression of its mRNA. We have demonstrated that *um02592* encodes a functional tartronate semialdehyde reductase (Tsr1), which showed dual specificity to cofactors NAD^+^ and NADP^+^ and strong substrate specificity and enantioselectivity for _D_-glycerate. Improved glycerol assimilation in GUM1 was associated with elevated expression of *tsr1* mRNA and this could be phenocopied by over-expression of the gene. Glycolipid accumulation was reduced by 45.2% in the knockout mutant whereas introduction of an extra copy of *tsr1* driven by the *glyceraldehyde phosphate dehydrogenase* promoter increased it by 40.4%.

**Conclusions/Significance:**

Our results demonstrate that tartronate semialdehyde reductase (TSR) plays an important role in glycerol assimilation in *U. maydis* and defines a novel target in genetic engineering for improved conversion of glycerol to higher value products. Our results add significant depth to the understanding of the glycerol metabolic pathway in fungi. We have demonstrated, for the first time, a biological role of a eukaryotic TSR.

## Introduction

**Figure 1:**
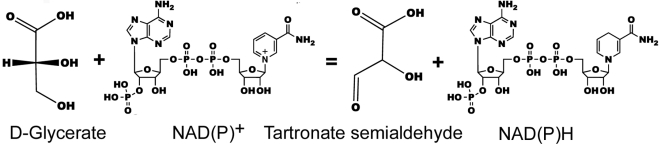
Tsr1-catalyzed reactions.

β-hydroxyacid dehydrogenases are a structurally conserved family of enzymes that catalyze NAD^+^ or NADP^+^ dependent oxidation of specific β-hydroxyacid substrates [1]. Tartronate semialdehyde reductase, or 2-hydroxy-3-oxopropionate dehydrogenase (TSR, EC 1.1.1.60), is a member of the β-hydroxyacid dehydrogenases family that catalyze the reversible reaction of tartronic semialdehyde to glycerate in the final stage of glycerate biosynthesis (Figure 1) [2], [3]. To date, only two bacterial TSRs have been characterized at the molecular and biochemical levels even though related sequences can be found in the genomes of archaea, fungi, plants and animals [4]. A β-hydroxyisobutyrate dehydrogenase was speculated to play a role in the utilization of polyhydroxybutyrate as a carbon source in *Haemophilus influenzae* [1]. However, the biological functions of TSRs remain unclear.

Glycerol is a by-product of biodiesel production, which accounts for about 10% of the triglyceride mass. It is predicted that glycerol may become heavily over-supplied when the production of biodiesel takes off [5], [6]. Currently, bioconversion targets of glycerol include 1,3-propanediol, dihydroxyacetone, ethanol, succinate, propionic acid, glyceric acid, citric acid, hydroxypyruvic acids, polyhydroxyalcanoate, pigments and biosurfactants [7], [8]. Except for 1,3-propanediol, product yield and conversion efficiency are poor in general. Therefore, identification of the key steps limiting the catabolism of glycerol will have a major impact on improving bioconversion efficiency of glycerol. In *Saccharomyces cerevisiae*, co-coordinated over-expression of glycerol dehydrogenase (Gcy), dihydroxyacetone kinase (Dak) and glycerol transporter (Gup1) have been shown to improve conversion of glycerol to ethanol by 3.4 folds over the wild-type strain, Nevertheless, only 2.4 g/l ethanol was produced out of 16.4 g glycerol consumed [8].

To the best of our knowledge, there has been no prior report on the use of *Ustilago maydis* for the bioconversion of glycerol. *U. maydis* has long been known to be an efficient producer of glycolipids, which are mainly composed of ustilagic acids (UA) and mannosylerythritol lipids (MEL) [9], [10], [11] that exhibit good surfactant properties [12]. With high biodegradability, mild production conditions and low toxicity, biosurfactants have applications in food processing, environmental protection, crude-oil recovery and drug delivery [13]. With comprehensive molecular tools and genetic and genomic resources [14], [15], [16], [17], [18], [19], [20], *U. maydis* would be an excellent host for the studies of glycerol catabolism. Here we report a T-DNA tagging strategy for fast identification of genes that improve the production of glycolipids in *U. maydis* using glycerol as the sole carbon source.

## Results

### Identification of glycerol utilization mutants

**Figure 2:**
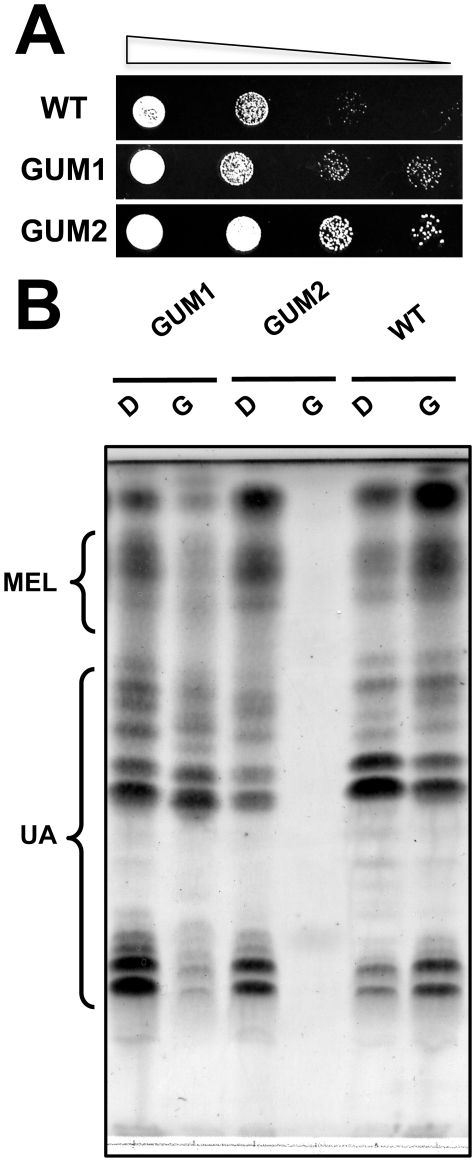
Phenotypes of GUM1 and GUM2. **A**. Screening for glycerol utilization mutants. Cultures of selected mutants were normalized to 1.0 OD_600_ and spotted on MinG agar plate in 10-fold serial dilutions. The photo was taken after incubation at 28°C for 2 days. **B**. Glycolipid profiles of WT, GUM1 and GUM2 in glucose or glycerol-containing medium. The MEL and UA species are indicated on the left. WT: wild-type strain (*U. maydis* L8 strain); D: dextrose as carbon source; G: glycerol as carbon source.

To determine whether *U. maydis* is suitable for the study of glycerol bioconversion, glycolipid-producing strain L8 was tested for the production of glycolipids in various media with glycerol as the sole carbon source. Synthetic minimal medium with glycerol (MinG) was found competent in producing both UA and MEL. We assumed that mutants with enhanced glycerol utilization capability would grow faster than most other colonies in the background. We created a random insertional mutants library of *U. maydis* L8 strain by the *Agrobacterium tumefaciens*-mediated transformation (ATMT) technique, and screened approximately 20,000 T-DNA insertional mutants on plates with glycerol as the sole carbon source. Twenty-four colonies that appeared significantly larger in size were selected and the screening was repeated in MinG agar medium by comparing the growth rates of the cells under various dilutions. As shown in Figure 2A, two best mutants showing clearly faster growth were selected and named as Glycerol Utilization Mutants No. 1 and 2 (GUM1 and GUM2). When cultured in glycerol-containing medium, GUM2 was severely defective in secretion of glycolipids while GUM1 largely retained the glycolipids profile found in the wild-type strain (WT) using either glucose or glycerol as carbon source (Figure 2B). GUM1 showed no obvious change in cell morphology and growth rate when cultured in glucose-containing medium (data not shown). Hence, we focused on the characterization of GUM1 in this report.

### Characterization of GUM1 and GUM2

**Figure 3:**
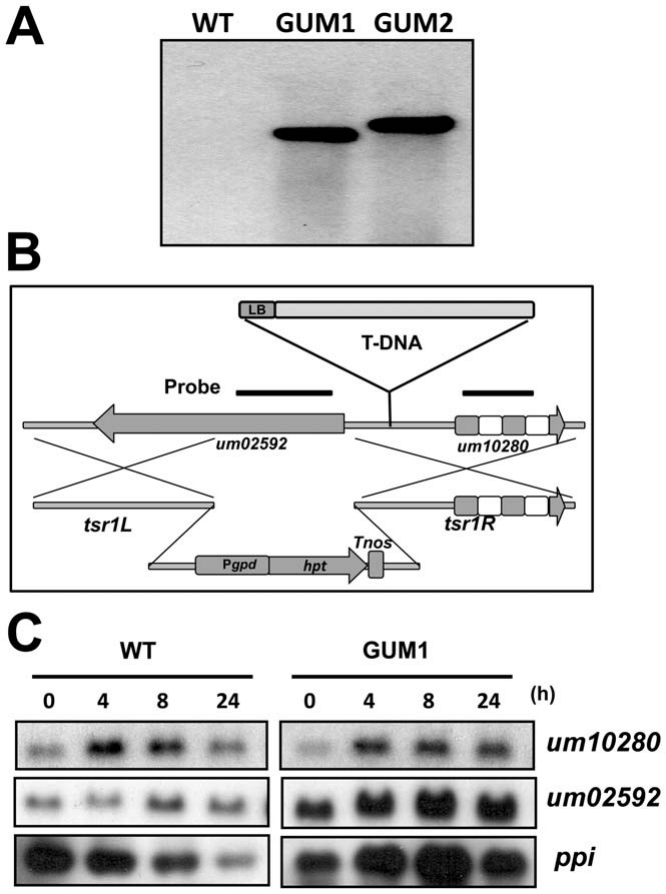
Characterization of GUM1. **A**. Southern blot analysis of GUM1 and GUM2. Genomic DNA (10 µg) of WT, GUM1 and GUM2 were digested with *Bam*HI and probed with a digoxigenin-labeled *hpt* DNA fragment. **B**. Schematic illustration of T-DNA insertion site in GUM1. LB: left border of T-DNA; P*gpd*: *gpd* promoter; *hpt*: coding sequence of Hygromycin resistance gene; *tsr1L* and *tsr1R*: homologous regions used for knockout of *tsr1*. **C**. Expression of *um10280* and *um02592*. WT and GUM1 cells were shifted to YPG medium and harvested after the time indicated (hours), and mRNAs were detected using the probes as illustrated in Figure 2B. Hybridization with *ppi* probe was used as the control for constitutive expression.

Southern blot analysis of GUM1 and GUM2 showed that both mutants contained a single-copy T-DNA integrated into the genome (Figure 3A). The T-DNA-tagged sequences were obtained by inverse PCR. BLASTn search showed that GUM1 contained a T-DNA inserted between the genomic locus of UM02592 and UM10280 (MIPS *Ustilago maydis* DataBase, MUMDB, with GenBank accession number XM_753646 and XM_753647 respectively), 236 bp upstream of the UM02592 locus and 274 bp upstream of UM10280 (Figure 3B). Northern blot hybridization showed that *um02592* mRNA was expressed at higher level in GUM1 compared with WT even when cultured in glucose-containing medium, whereas *um10280* mRNA expression was not significantly different from WT (Figure 3C). Noticeably, *um02592* appeared to be inducible by glycerol in GUM1 (Figure 3C). These results suggest that *um02592* is likely to be involved in the observed phenotype of GUM1.

### Molecular analysis of *um02592* gene

**Figure 4:**
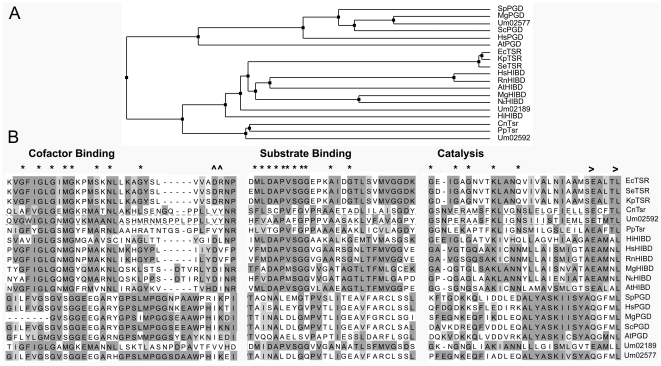
Sequence analysis of β-hydroxyacid dehydrogenases. **A**. Phylogenic tree of selected β-hydroxyacid dehydrogenases. Sequence alignment and phylogenic tree was created with Jalview 2.5 program [36] using the average distance method with BLOSUM62 matrix. The sequences and origins can be found in the following Protein Identifiers from GenBank: 145698315 (EcTSR, *Escherichia coli* str. K-12 substr. MG1655); 16766546 (SeTSR, *Salmonella enterica* subsp. enterica serovar Typhimurium str. LT2); 152972053 (KpTSR, *Klebsiella pneumoniae* subsp. pneumoniae MGH 78578); 57226089 (CnTsr, putative TSR of *Cryptococcus neoformans* var. neoformans JEC21); 46098529 (Um02592, *Ustilago maydis* 521); 242207395 (PpTsr, putative Tsr of *Postia placenta* Mad-698-R); 16272945 (HiHIBD, *Haemophilus influenzae* Rd KW20); 23308751 (HsHIBD, *Homo sapiens*); 83977457 (RnHIBD, *Rattus norvegicus*); 39952089 (MgHIBD, *Magnaporthe grisea* 70-15); 32411867 (NcHIBD, *Neurospora crassa* OR74A); 18415593 (AtHIBD, *Arabidopsis thaliana*); 19111887 (SpPGD, *Schizosaccharomyces pombe*); 40068518 (HsPGD, *Homo sapiens*); 145611724 (MgPGD, *Magnaporthe grisea* 70-15); 458910 (ScPGD, *Saccharomyces cerevisiae*); 15232888 (AtPGD, *Arabidopsis thaliana*); 46098078 (Um02189, *Ustilago maydis* 521) and 71014537 (Um02577, *Ustilago maydis* 521). **B**. Conserved motifs. *, ∧ and > indicate functionally important residues [4].

Based on the sequence information on *U. maydis* 521 strain (MUMDB), the ORF of Um02592 was amplified by PCR from the genomic DNA of L8 strain. It contains two silent nucleotide substitutions (G93A and G543A) and the third (A234T) substitution converts the 78^th^ residue from glutamic acid to aspartic acid. A BLASTp search of Um02592 (acc. no. EAK83762) revealed that it shares 44% identity to a putative β-hydroxyacid dehydrogenase from *Cryptococcus neoformans* (acc. no. EAL21888) and a long list of other related β-hydroxyacid dehydrogenases from bacteria, fungi, plants and animals, including 6-phosphogluconate dehydrogenases (PGD), 3-hydroxyisobutyrate dehydrogenases (HIBD) and tartronate semialdehyde reductases (TSR). We selected a few representative proteins from each class, which included at least one biochemically characterized enzyme, to analyze the phylogenic relationship (Figure 4A; Support information Figure S1). It was obvious that Um02592 and two other related sequences of fungal origin (CnTsr and PpTsr) belonged to a distinct clad and were most divergent from the PGD clad. However, it was not possible to clearly distinguish Um02592 from the HIBD and bacterial TSR clads. Since the *U. maydis* genome contains another protein (Um02577) more related to the PGDs and HIBDs (Um02189), we predicted that Um02592 is more likely to be a homolog of TSR. A detailed examination of Um02592 revealed that it contains the majority of residues found in the signature sequences of β-hydroxyacid dehydrogenases, including the cofactor binding, substrate binding and catalytic domains [4] (Figure 4B). However, we noticed that Um02592 contains a high degree of divergence in the substrate-binding and catalytic domains, which are highly conserved between TSRs and HIBDs.

### Biochemical properties of Tsr1

To determine the biochemical function of Um02592, we expressed the cDNA in *E. coli* and purified the recombinant enzyme as a hexa-histidine-tagged protein. Soluble recombinant protein (rTsr1) was produced when the induction temperature was lowered to 25°C. The protein was readily enriched more than 8-fold to higher than 95% homogeneity by a single cycle of affinity chromatography in a Ni-chelating column (Support information Table S1). The purified enzyme exhibited a single dominant band on SDS-PAGE, migrating with an apparent molecular weight of about 40 kDa (Support information Figure S2A). This in good agreement with the predicted size of 41 kDa and protein band was able to cross-react with anti-hexa-histidine antibodies (Support information Figure S2B). Gel filtration result showed that rTsr1 has a molecular weight of 85 kDa in its natural state, suggesting that it exists as a dimer in aqueous solution (Support information Figure S2A and 2C). The purified rTsr1 was highly unstable in Tris-Cl buffer (pH 8.0) and rapidly precipitated during storage (<12 h at 4°C). However, enzyme stability and solubility could be improved in phosphate buffer at pH 7.0 (Support information Figure S3D).

**Figure 5:**
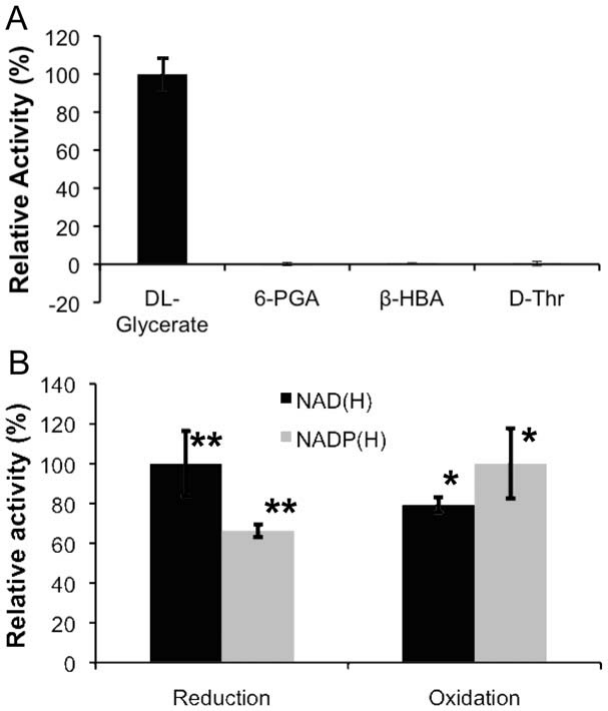
Tsr1-catalyzed reactions. **A**. Oxidation of various substrates. β-HBA: β-hydroxybutyric acid; 6-PGA: 6-phosphogluconate acid; D-thr: _D_-threonine. **B**. Effects of cofactors on reduction and oxidation reactions. Standard deviation (SD) derived from triplicates. * and ** indicate significant differences in the group (p<0.05 and 0.001 respectively) based on one-way ANOVA test.

The purified rTsr1 was able to oxidize _DL_-glyceric acid using NAD(P)^+^ as cofactor. The test of four different β-hydroxyacid substrates showed that rTsr1 was highly specific to glyceric acid and exhibited little activity towards 6-phosphogluconic acid, β-hydroxybutyric acid and _D_-threonine (Figure 5A). Although NAD^+^ could also be used as a cofactor, the enzyme showed approximately 26% lower activity compared with NADP^+^ in the oxidation of _DL_-glyceric acid (Figure 5B). rTsr1 showed an optimal activity at 40°C and pH 8.5, although it had better stability around pH 7.0 (Support information Figure S3A, 3B and 3D). An abrupt drop in enzyme stability and activity was observed at pH 6.5, presumably due to precipitation of the enzyme as this coincides with its predicted isoelectric point (Support information Figure S3C and S3D). In the reduction reaction, rTsr1 showed an optimal pH of 5.5 (Support information Figure S3B). rTsr1 activity seemed to require metal ions as cofactor as EDTA severely inhibited its activity (Support information Table S2).

Using non-linear regression method, the Michaelis constants *K_m_* and *V_max_* of rTsr1 on _D_- and _L_-glycerate was determined. The enzyme had a *K_m_* of 17.7 mM and 123.2 mM with _D_-glycerate and _L_-glycerate respectively, indicating a much higher affinity to _D_-glycerate (Table 1). In the reduction reaction with tartronic semialdehyde and NADH as the substrate and cofactor respectively, the *K_m_* was dramatically reduced (0.19±0.03 mM) (Table 1). This suggests that the metabolic flow to glycerate production is favored under normal conditions.

**Table 1:** Kinetic parameters of rTsr1^a^.

	Substrate	*K_m_* b	*V_max_* (µmol/min/mg)
**Oxidation**			
	_D_-Glycerate	17.7±2.3 mM	1.14±0.15
	_L_-Glycerate	123.2±21.8 mM	0.03±0.01
	β-NADP^+^	0.30±0.04 µM	0.93±0.07
**Reduction**			
	Tartronic semialdehyde	0.19±0.03 mM	0.17±0.03
	β-NADH	14.29±3.1 µM	5.07±0.39

^*a*^The purified recombinant enzyme (0.1 µg/assay) was incubated with various substrates in a reaction system at 40°C and repeated in triplicates.

^*b*^ *K_m_* values for each substrate were determined with six concentrations of substrate and six concentrations of cofactor. For oxidation reaction, *K_m_* for NADP^+^ was calculated from reactions using _D_-glycerate as substrate. For reduction reaction, *K_m_* for NADH was calculated from reactions using tartronic semialdehyde as substrate.

### Over-expression and gene deletion of *tsr1*

**Figure 6:**
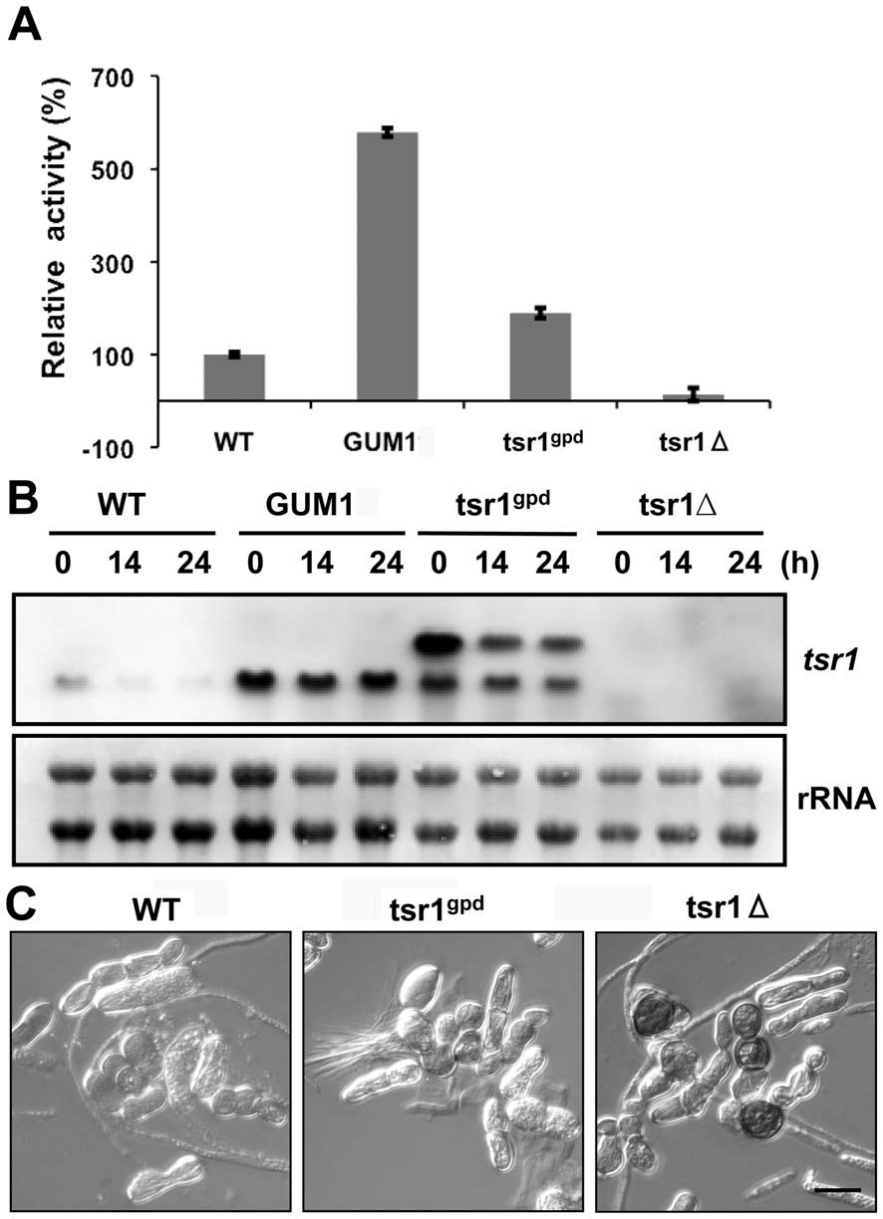
Effects of *tsr1* knockout and over-expression. **A**. Relative TSR activity in various strains. Tsr1 activity was assayed using _DL_-glycerate as substrate and NADP^+^ as cofactor according to the standard enzyme assay method. The activity of WT is set at 100%. tsr1Δ and tsr1^gpd^ are the knockout and over-expression strains in L8 background, respectively. Tsr1 activity was assayed in triplicates. **B**. Expression of *tsr1* mRNA in various strains. Strains were cultured in YPD medium to late exponential phase (time zero) before shifted to MinG medium for the duration indicated. The blot was probed with digoxigenin-labeled *tsr1* cDNA. In tsr1^gpd^, the upper band is the overexpressed *tsr1-egfp* mRNA. The rRNA bands were stained with Methylene Blue. **C**. Cell morphology and glycolipids accumulation. Cells of WT, tsr1^gpd^ and tsr1Δ strains that were cultured for 14 days in MinG medium. The needle-like or fibrous material around the cells is UA. The scale bar represents 10 µm.

To confirm that *tsr1* is involved in glycerol catabolism in *U. maydis*, gain-of-function (tsr1^gpd^) and loss-of-function (tsr1Δ) mutants were generated using ATMT technique. As expected, tsr1Δ strain displayed a 45.2% reduction in glycolipids production and the utilization of glycerol was also reduced. The opposite effect was observed with the tsr1^gpd^ strain, which had an approximate 40% increase in glycolipids accumulation (Table 2). Analysis of Tsr1 activities of the cell extract of the strains that were cultured for 24 hours in glycerol-containing medium showed the tsr1Δ strain had essentially abolished Tsr1 activity whereas the tsr1^gpd^ strain had approximately 100% higher activity over the WT (Figure 6A). Northern blot analysis confirmed the enhanced mRNA expression in the tsr1^gpd^ strain and total absence of expression in the tsr1Δ strain (Figure 6B). tsr1Δ cells showed no obvious abnormality except that accumulation of black pigment was observed when cultured in MinG medium (Figure 6C). These results confirm that *tsr1* play a major role in the bioconversion and assimilation of glycerol in *U. maydis*.

**Table 2:** Effects of knockout (tsr1Δ) and over-expression (tsr1^gpd^) strains of *tsr1* a.

	Cell Biomass	Residual Glycerol	Glycolipids
WT	5.96±0.43	16.34±1.44	14.88±2.15*
tsr1^gpd^	6.41±0.30	15.78±0.96	20.89±3.02*
tsr1Δ	6.40±0.33	19.10±0.55*	8.15±0.13**

^*a*^The units used are in g/l.

*and ** indicate significant differences in the group (p<0.05 and 0.001, respectively) based on ANOVA test between WT and *tsr1*-mutant groups. Cells were fermented in MinG medium for 14 days.

### Restoration of glycolipids production by supplementation of glycerate

**Figure 7:**
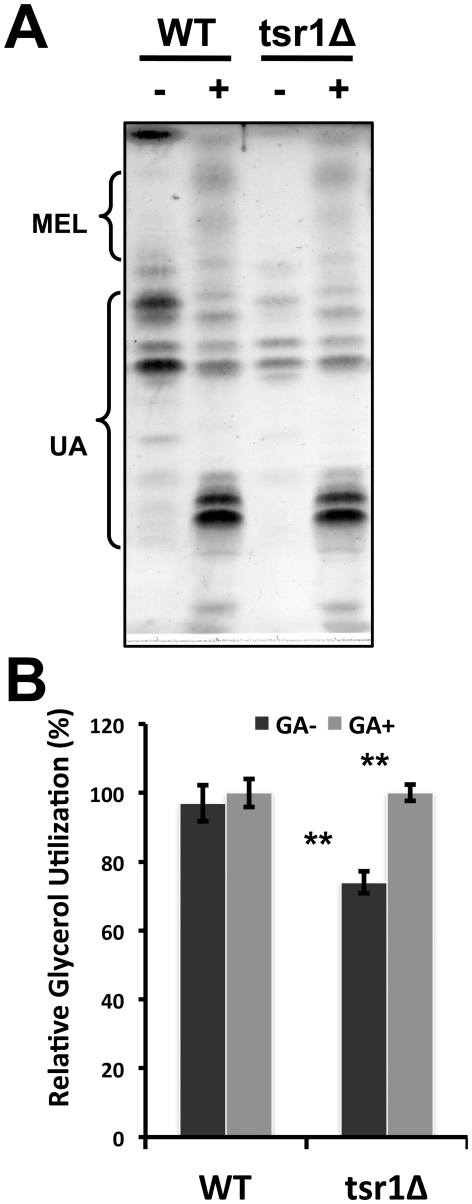
Effects of glycerate supplementation. **A**. Glycolipid profiles of WT and tsr1Δ. Single colonies of WT and tsr1Δ were cultured in MinG (10 g/l glycerol) with (+) or without (-) _DL_-glycerate (1%, v/v) at 30°C, 250 rpm for 5 days. **B**. Glycerol utilization in WT and tsr1Δ. Residual glycerol was quantified by HPLC after a 5-day culture in MinG medium and the relative glycerol utilization was calculated from three independent repeats. ** indicates statistical significance (p<0.001) by one-way ANOVA test.

Since the *loss-of-function* strain tsr1Δ produced considerably less glycolipids than WT in MinG medium, we explored to see whether this defect could be complemented with glycerate. As expected, WT and tsr1Δ showed comparable glycolipids profiles when _DL_-glycerate was supplemented to MinG medium (Figure 7A). As shown in Figure 7B, glycerol utilization in WT was only slightly improved by the supplementation of glycerate. On the other hand, glycerol utilization was restored to WT level in tsr1Δ when glycerate was supplemented. These results suggest that glycerate production catalyzed by Tsr1 is a rate-limiting step in the bioconversion of glycerol to glycolipids in *U. maydis*.

### Promoter analysis of *tsr1*

**Figure 8:**
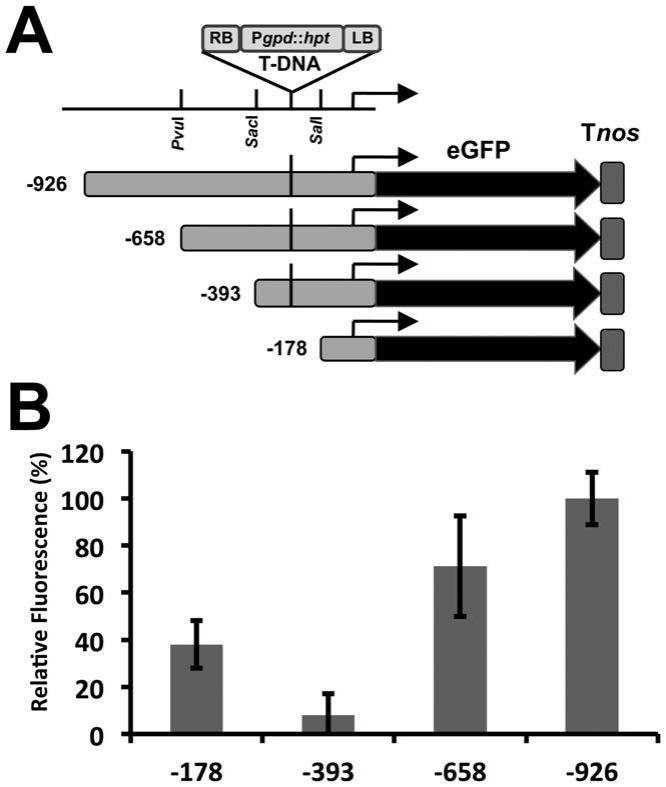
Deletion analysis of *tsr1* promoter. **A**. Schematic illustration of reporter constructs. The location of T-DNA insertion site in GUM1 is indicated. *Tnos*: transcriptional terminator of *A. tumefaciens nopaline synthase* gene. **B**. Relative fluorescence intensity of reporter constructs. The normalized fluorescence intensity of the -926 promoter was set at 100% and results were average of triplicates.

To investigate the transcriptional regulation of *tsr1*, eGFP [21] encoding sequence was fused to the upstream sequences of the Tsr1-encoding region (Figure 8A). The strongest fluorescence was observed in the longest promoter (-1 to -926) and shortening the promoter towards the 3′ region steadily reduced the promoter activity. Notably, inclusion of the sequence from -179 to -393, the region in which the T-DNA was inserted in GUM1, led to a nearly 9-fold decrease in promoter activity (Figure 8B). These results suggest that the region from -393 to -179 contain a negative *cis*-acting DNA element with an important role in the regulation of *tsr1* expression. A binding motif (AGGGG) for yeast Gis1p, a transcriptional factor with known functions in reprogramming of carbon metabolism in yeast [22], [23], was identified through scanning the above 214 bp sequence region using the YEASTRACT program (http://www.yeastract.com/formtfsbindingsites.php). Further studies on the -393 to -179 region should shed more light on the regulatory control of *tsr1* in *U. maydis*.

## Discussion

**Figure 9:**
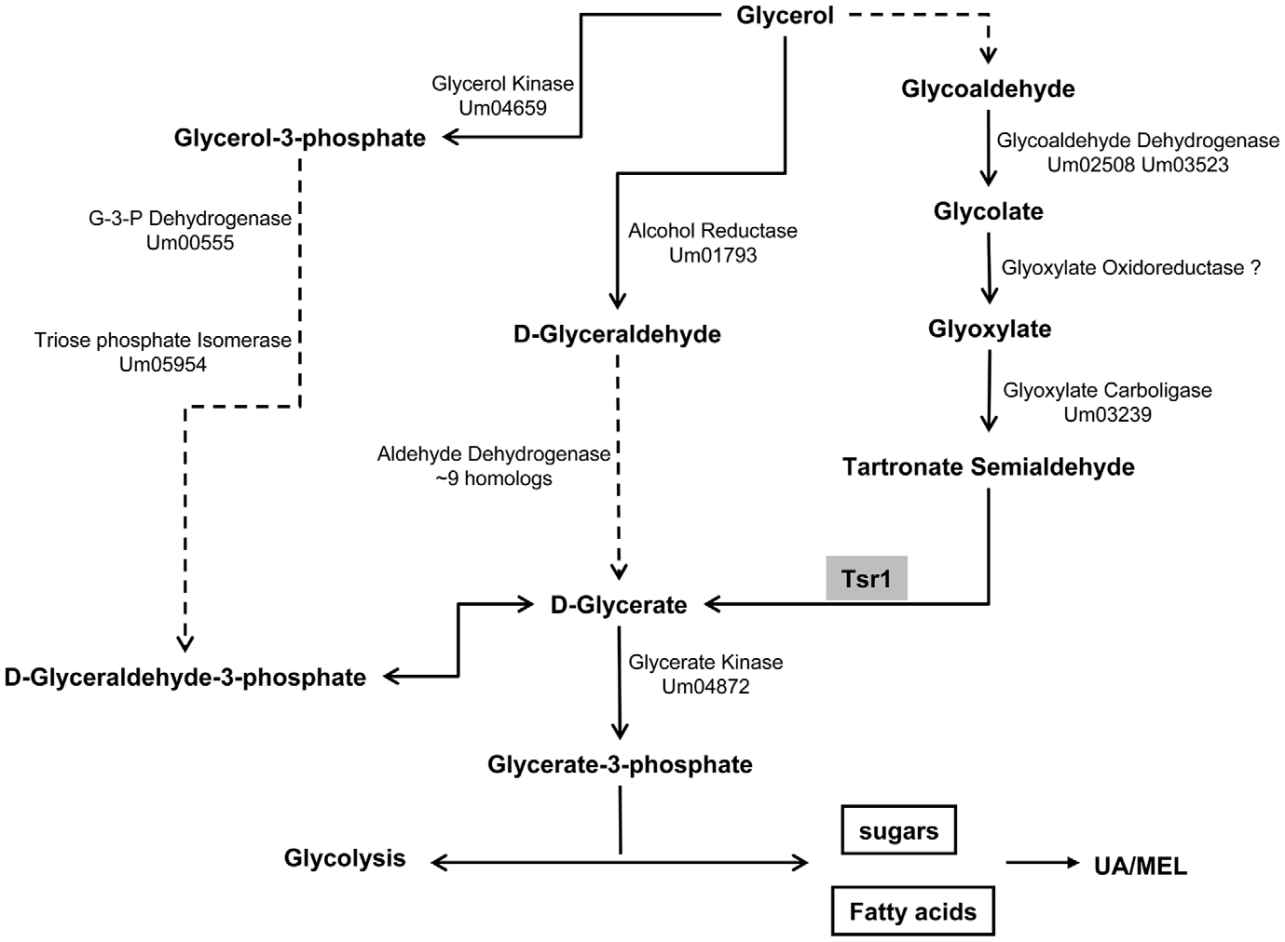
Model of glycerol metabilosm in *U. maydis*. The pathway is based on Tom et al. [24] and the enzymes for various steps are based on the current information in the KEGG DataBase (http://www.genome.jp/kegg/) and the MIPS *Ustilago maydis* Genome Database (http://mips.helmholtz-muenchen.de/genre/proj/ustilago/).

Currently, three glycerol metabolic pathways have been reported [24], one phosphorylative pathway [25], [26] and two oxidative pathways that are based on NAD(P)^+^-dependent glycerol dehydrogenases [27]. In this report, we have identified and characterized the first eukaryotic TSR and demonstrated its role in glycerol catabolism and production of glycolipids from glycerol (Figure 5, 6, S3, S4, Table 2). Our results support the proposal that Tsr1 defines a rate-limiting step in assimilation of glycerol leading to the production of glycerate, which is an important metabolic intermediate for the biosynthesis of sugars and fatty acids, the precursors of glycolipids. In principle, glycerate production may also be catalyzed by glyceraldehyde dehydrogenase. As the substrate of Tsr1, tartronate semialdedyhe can be produced by glyoxylate carboligase from glyoxylate, or from hydroxypyruvate by the hydroxypyruvate isomerase [4]. Putative homologs of these genes can be found in the *U. maydis* genome (Figure 9). Thus, over-expression of these enzymes might further enhance the assimilation and bioconversion rates of glycerol to glycolipids. It is very interesting to note that the product of Tsr1, glycerate, was able to improve glycerol utilization in tsr1Δ (Fig. 7B). One possible explanation for this is that glycerate is an inducer for the expression of certain genes in the alternative glycerol catabolic pathways (Figure 9).

The low sequence conservation of Tsr1 to either TSRs or HIBDs in the substrate-binding domain (Figure 4B) is quite surprising. The cofactor-binding domain in TSRs, HIBDs and PGDs share the same consensus with that of the NAD^+^-dependent D(-)-lactate dehydrogenase (D-LDH) from *Lactobacillus delbrueckii*, GXGXXGX_(17)_D, in which a change in the aspartate residue to alanine shifts cofactor selectivity from NAD^+^ to NADP^+^ [28]. We noticed that both Tsr1 and its homologs in two other related fungi lack an aspartate residue in the cofactor-binding motif (Figure 4B). Hence, Tsr1 from *U. maydis* would be a good enzyme target to elucidate the dual cofactor specificity to NAD^+^/NADH and NADP^+^/NADPH.

## Materials and Methods

### Strains, growth conditions and chemicals

*U. maydis* L8 is an *a1 b1* strain isolated from China [19]. The strain was cultured at 28°C in YPD broth (1% yeast extract, 2% peptone, 2% glucose) or on solid potato-dextrose agar (PDA). *Agrobacterium tumefaciens* AGL-1 [29] was grown at 28°C in either liquid or solid 2YT medium. ATMT of *U. maydis* was performed as described [19]. *Escherichia coli* strain XL10 was cultured in LB medium and used for routine plasmid manipulation while BL21(DE3) (Novagen, USA) was used for recombinant protein expression.

_DL_-glyceric acid was purchased from CP Chemicals (Japan) and _D_-glycerate, _L_-glycerate, β-hydroxybutyric acid, 6-phosphogluconate acid, _D_-threonine and other chemicals were from Sigma-Aldrich (USA). The tartronic semialdehyde was prepared by the enolization of lithium hydroxypyruvate in alkaline medium with a conversion rate of approximately 50% [30] and the concentration was determined by HPLC. For production of glycolipids, *U. maydis* was cultured in MinAB medium [31] with 50 g/l of glucose (MinD) or 50 g/l of glycerol (MinG) at 30°C with constant shaking (200 rpm).

### DNA constructs

Binary plasmids pTHPR1 has been described previously [19] and the structures of pEX1GPD-GUS, pEX1GPD-eGFP and pEX2tk can be found in Support information Figure S4.

Gene knockout construct pKOtsr1 was constructed by joining, in a single ligation, four DNA fragments, i.e., the 8668 bp *Nco*I-*Kpn*I-cut and dephosphorylated pEX2tk, the 1994 bp *Hin*dIII-*Spe*I-cut fragment containing the P*gpd::hpt::*T*_35S_* gene cassette, the 1577 bp *Nco*I-*Hin*dIII-cut 5′ flanking sequence and the 2688 bp *Spe*I-*Kpn*I-cut 3′ flanking sequence of *um02592* gene. Oligos Tsr1L-Nf and Tsr1L-r were used for PCR of the 5′ flanking region while Tsr1R-f and Tsr1R-Br were used for PCR of the 3′ flanking region of *um02592*. Candidate constructs were identified by colony PCR, restriction digestion mapping and further confirmation by sequencing. ATMT was used to generate transformants and fungal colony PCR was used to identify candidate knockout strains, which were further confirmed by Southern blot analysis.

For overexpression of *tsr1* in *U. maydis*, oligos Tsr1-Bsf and Tsr1-Br were used for RT-PCR. The *Bsp*HI-*Bam*HI double-digested PCR product was ligated to *Nco*I-*Bam*HI double-digested pEX1GPD-EGFP to create pEX1GPD-Tsr1EGFP, and *gain-of-function* mutant tsr1^gpd^ was generated by ATMT technique. All sequence information of the oligos used can be found in Support information Table S3.

### Analysis of T-DNA insertion site

*Kpn*I or *Spe*I digested genomic DNA of GUM1 was self-ligated before being used as templates for inverse PCR [32] with IP1 and IP2 as primers. Semi-nested PCR was performed using a 100-fold dilution of the inverse PCR product as template and IP1 and RBtail1 as primers. PCR was carried out using Taq DNA polymerase (Qiagen, Germany) in a PTC-200^TM^ Programmable Thermal Controller (BioRad, USA). PCR products were gel-purified (Qiagen kit), cloned into pGEM-T Easy vector (Promega, USA) and sequenced using BigDye Terminator sequencing method (ABI, USA).

### Quantification of glycolipids and glycerol

Extraction and thin-layer chromatographic identification of glycolipids were performed essentially as described previously [16]. For quantification of glycolipids, samples were diluted 10 times and separated in a Silica C18 Column (Φ5 µm, 4.6×150 mm) (Waters, USA) using methanol and 0.05% formic acid as the mobile phase. The samples were run at a flow rate of 1.0 ml/min. A column heater (Shimadzu, Japan) was used to maintain the running temperature at 45°C. Glycolipids were detected at 40°C with an evaporative light scattering (ELSD) detector (Shimadzu, Japan). Data was acquired and analyzed with LCsolution (ver1.2) (Shimadzu, Japan). The hexane-purified glycolipids from *U. maydis* L8 strain were used as the control and the amount of glycolipids was the average of triplicates.

For quantification of glycerol, samples (10 µl) were injected and run in an Aminex 87H column (Φ5 µm, 7.0×300 mm) (Bio-Rad, USA) at a flow rate of 0.7 ml/min using 5 mM sulfuric acid as the mobile phase. The column was maintained at 50°C and glycerol was detected with a Refractive Index Detector (RID, Shimadzu, Japan).

### Northern blot analysis

Cells were cultured in YPD medium at 28°C until late logarithmic phase and collected by centrifugation. The cells were washed with sterile water and resuspended in an equal volume of yeast extract-peptone-glycerol (YPG) medium. Total RNA was extracted at various time points in YPG medium and the concentrations were determined with a Nanodrop photospectrometer (Nanodrop Technologies, USA). A 553-bp internal fragment of the gene was amplified by PCR using primers Tsr1f and Tsr1r and used as a probe for Northern analysis of *tsr1* expression. Expression of *um10280* mRNA was probed with a 416-bp fragment, which was amplified by PCR using primers Vas1f and Vas1r. Northern hybridization was performed as described [33]. The hybridization membrane was striped and re-hybridized against the *peptidylprolyl isomerase* encoding gene *ppi* (um03726) [34]. The 456 bp *ppi* probe of was amplified by PCR using primers Ppif and Ppir.

### Expression and purification of recombinant Tsr1

To express Tsr1 in *E. coli*, *tsr1* was amplified from total RNA of wild-type L8 strain by RT-PCR using oligos Tsr1-Ef and Tsr1-Xr. The *Eco*RI/*Xho*I double-digested PCR product was ligated to the similarly digested pET-28a (Novagen, USA) to create pETTsr1, which was subsequently transformed into *E. coli* strain BL21(DE3). Kanamycin and Chloramphenicol resistant transformants were cultured overnight at 37°C in 25 ml Luria broth (LB) supplemented with 25 µg/ml Kanamycin and 34 µg/ml Chloramphenicol. The culture was scaled up until OD_600_ reached 0.7 unit before induction was initiated by adding isopropyl-D-thiogalactoside (IPTG) to a final concentration of 0.4 mM. After approximately 20 h induction at 25°C, the culture was harvested, washed with water and the cell pellet was suspended in 15 ml buffer A (50 mM Tris-Cl, 0.3 mM PMSF, 1 mM DTT, pH 7.0). Cells were disrupted by sonication and the insoluble materials were sedimented by centrifugation at 4°C, 14,000 g for 30 min. Protein was purified with an ÄKTA^TM^ Purifier (GE Healthcare, USA) at 4°C using a HisTrap_FF column (5 ml), which was equilibrated with buffer A supplemented with 500 mM NaCl and 20 mM imidazole. The His-tag fusion protein was eluted with buffer A supplemented with 500 mM NaCl and 200 mM imidazole. The concentration of protein was quantified using the Bradford method [35].

To prepare cell extract for TSR activity assay, cells were harvested after 24 h culture in MinG medium, resuspended in PBS buffer (pH 7.4) supplemented with 5 mM DTT and 1 mM PMSF. Total protein was recovered after sonication and centrifugation. The protein contents were determined by the Bradford method [35] and normalized to 1.0 mg/ml.

### Enzyme assay

TSR activity was determined as described previously [4] with some modifications. Enzyme preparation (10 µl) was added to 190 µl pre-warmed (40°C) reaction mixture consisting of 50 mM glycine, 100 µM β-NAD^+^ or β-NADP^+^, and 2 mM _DL_-glyceric acid (pH 8.5). The absorbance at 340 nm was monitored using a Tecan Infinite® M200 plate reader and data was collected using I-Control^TM^ software (Tecan, Germany). The kinetic parameters were calculated as described previously [3]. One unit of activity is defined by the production of 1 µmol of NADH or NADPH per minute at 40°C. Enzyme activity was calculated using following equation: Tsr1 unit  =  v * s/(ε * l), wherein *v* is the volume of reaction system (200 µl), *s* is the increase of OD_340_ value per minute, *ε* is the molar extinction coefficient of NADH or NADPH at 340 nm (*ε_NADH_*  = 6220 µl/µmol cm; *ε_NADPH_*  = 6270 µl/µmol cm), *l* is the depth of a 200 µl reaction system in one well (*l*  = 0.6 cm in the Nunc^TM^ transparent 96-well plate).

### Enzyme stability and optima

The optimum temperature and pH for activity of rTsr1 were determined by varying the assay temperature and pH values in the reaction mixture, respectively. Thermal stability was obtained after pre-incubation of rTsr1 at different temperature for 30 min. pH stability was investigated after pre-incubation of rTsr1 in various pH buffers for 30 min before the assay at pH 7.0. Pre-treated samples were centrifuged at 4°C, 14, 000 g for 5 min to remove the denatured protein before the assay. In all cases, overlaps were obtained when buffers were changed to correct the spurious buffer effects.

### Substrate and cofactor specificity

Activity of purified rTsr1 (1.0 µg/assay) was assayed using various substrates, i.e., _DL_-glycerate (25 mM), _D_-glycerate (25 mM), _L_-glycerate (25 mM), 6-phosphogluconic acid (0.5 mM), β-hydroxybutyric acid (0.5 mM) and _D_-threonine (0.5 mM). NAD^+^ and NADP^+^ (10 mM) were supplemented individually to determine the preference for cofactor with _DL_-glycerate as the substrate.

### Analysis of promoter activity

To generate the 926 bp promoter fusion construct with enhanced green fluorescence protein encoding gene, the upstream sequence of *tsr1* was amplified using WT genomic DNA as template and oligo pair Vas1r/Ptsr1-Nr. The PCR products were end-blunted with T4 DNA polymerase, digested with *Nco*I and ligated with the *Pme*I/*Nco*I double-digested pEX1GPD-eGFP. To generate 658 bp, 393 bp and 178 bp promoter-eGFP gene cassettes, the above 926 bp PCR product was double-digested with *Nco*I and *Pvu*I, *Sac*I or *Sal*I, respectively (Figure 7A). All transformants were obtained by ATMT technique. To minimize positional effect of reporter gene expression, at least 1000 transformants on the membrane were collected to create a mixed population of transformants, which were cultured and assayed for the strength of GFP florescence. Sporidia culture at mid-exponential phase was pelleted by centrifugation and thoroughly washed with water. After diluted to 0.2 OD_600_ in MinG medium, the cells were cultured at 30°C for 2 h. An aliquot (100 µl) was loaded to a well in a 96-well plate. Flat transparent plates (Nunc, USA) and MicroFLUOR round-bottom plates (Dynatech, USA) were used for determination of cell optical density and GFP fluorescence intensity in a Tecan Infinite® M200 plate reader (Tecan, Germany), respectively. Cell optical density was read at 600 nm while GFP fluorescence was measured with the excitation and emission wavelength set at 488 nm and 508 nm, respectively. The fluorescence intensity was normalized against the cell density (OD_600_).

### Light microscopy

*U. maydis* cells were observed with a Nikon Eclipse 80i microscope equipped with CFI Plan Apochromat objectives (Nikon, Japan), and images were acquired with a Nikon DS camera and Nikon ACT-2U software.

## Supporting Information

Figure S1 **Full alignment of selected β-hydroxyacid dehydrogenases.** GenBank ID and origins of the proteins can be found in Figure 4.
(TIF)

Figure S2 **Expression and purification of hexa-histidine-tagged Tsr1.** **A**. SDS-PAGE of rTsr1. Proteins were separated on a 10% SDS-PAGE and stained with Coomassie brilliant blue R-250. M: low range protein molecular weight marker (Bio-Rad). Molecular weights are shown on the left (kDa); WC: whole cell extract; CE: crude enzyme; Eluate, fractions eluted using 0.2 mM imidazole. **B**. Western blot analysis of rTsr1. Proteins in (A) were blotted, hybridized against mouse anti-His antibodies (GE Healthcare). Biotinylated goat anti-mouse IgG (Millipore Chemicon, USA) was used as secondary antibody and detection was performed with CPD-star (Roche Diagnosis) after binding with Streptavidin-AP conjugate (Roche Diagnosis). **C**. Gel filtration chromatography of rTsr1. Standard proteins (GE healthcare) and the purified enzyme were passed through a Superdex 200 gel filtration column (16 × 300 mm), and the relationship between molecular mass and *Ve/Vo* (ratio of exclusion and void volume of gel matrix) was determined. The arrowhead-labeled standard proteins are as followed: 1. Ferritin, 440 kDa; 2. BSA, 67 kDa; 3. β-lactoglobulin, 35 kDa; 4. Ribonuclease, 13.7 kDa.
(TIF)

Figure S3 **Biochemical characterization of rTsr1.** **A**. Optimum temperature. **B**. Optimum pH. Oxidation and reduction reaction are marked as solid and dashed lines, respectively. **C**. Thermal stability. **D**. pH stability. Standard deviation (SD) derived from triplicates.
(TIF)

Figure S4 **Schematic illustration of T-DNA regions of cloning vectors.** **A**. pEX1GPD-GUS. **B**. pEX1GPD-eGFP. **C**. pEX2tk. LB: left border of T-DNA; RB: right border of T-DNA; P*gpd*: promoter of *gpd*; *hpt*: Hygromycin resistance gene; *gus*: β-glucuronidase gene; eGFP: enhanced Green Fluorescence Protein; T*nos*: terminator of *nopaline synthase* gene of *A. tumefaciens*; T*_35S_*: terminator of *Cauliflower mosaic virus 35S* gene; T*_tef_*: terminator of the *translational elongation factor 1* gene of *Ashbya gosspii*. All vectors have the same pPZP200 backbone.
(TIF)

Table S1 **Summary for purification of rTsr1.**
(RTF)

Table S2 **Effects of metal ions, chelating agent, and reducing and oxidative agents.**
(RTF)

Table S3 **Oligonucleotides used.**
(RTF)
