# Supplementary figures and images for: Tartronate Semialdehyde Reductase Defines a Novel Rate-Limiting Step in Assimilation and Bioconversion of Glycerol in Ustilago maydis
Source: PLoS One. 2011 Jan 31;6(1):e16438. doi: 10.1371/journal.pone.0016438 (PMC3031564; doi:10.1371/journal.pone.0016438)

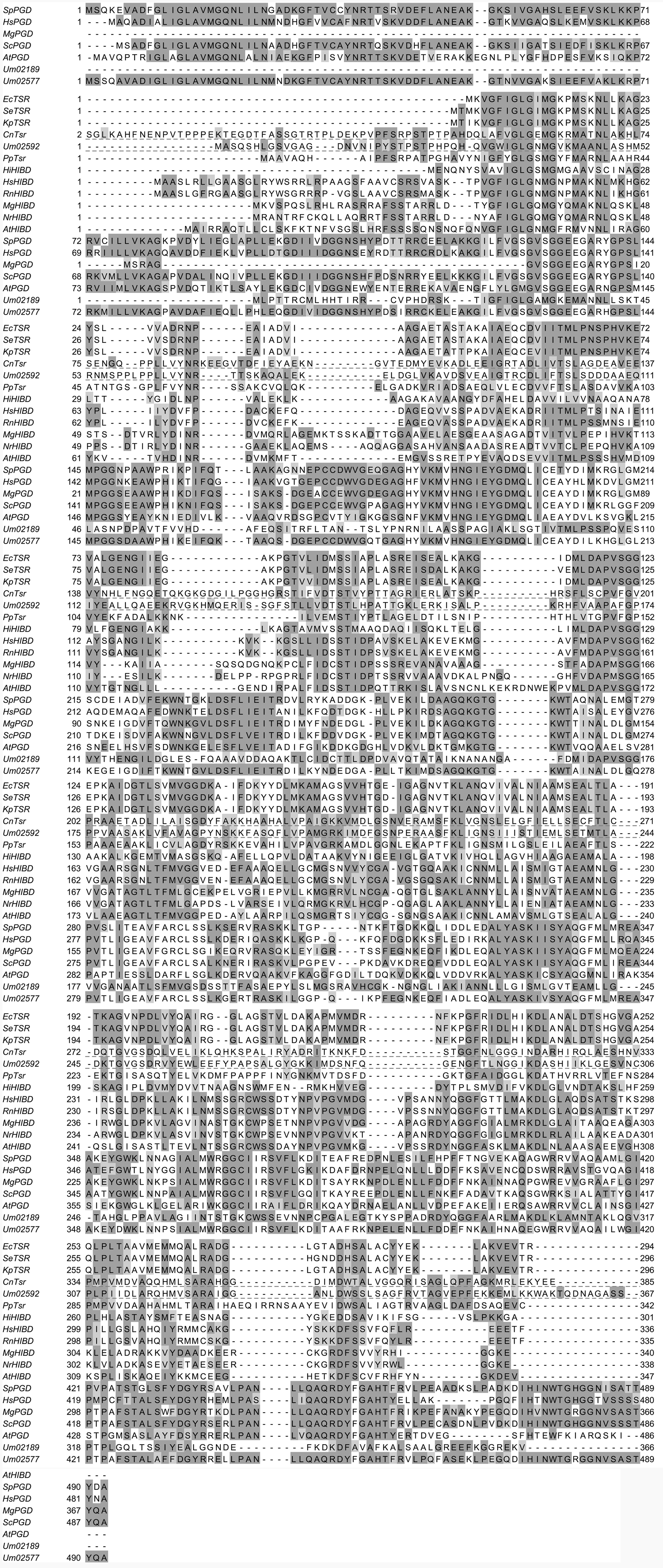

Supplement: Figure S1 — Full alignment of selected β-hydroxyacid dehydrogenases. GenBank ID and origins of the proteins can be found in Figure 4. (TIF) [file pone.0016438.s001.tif]

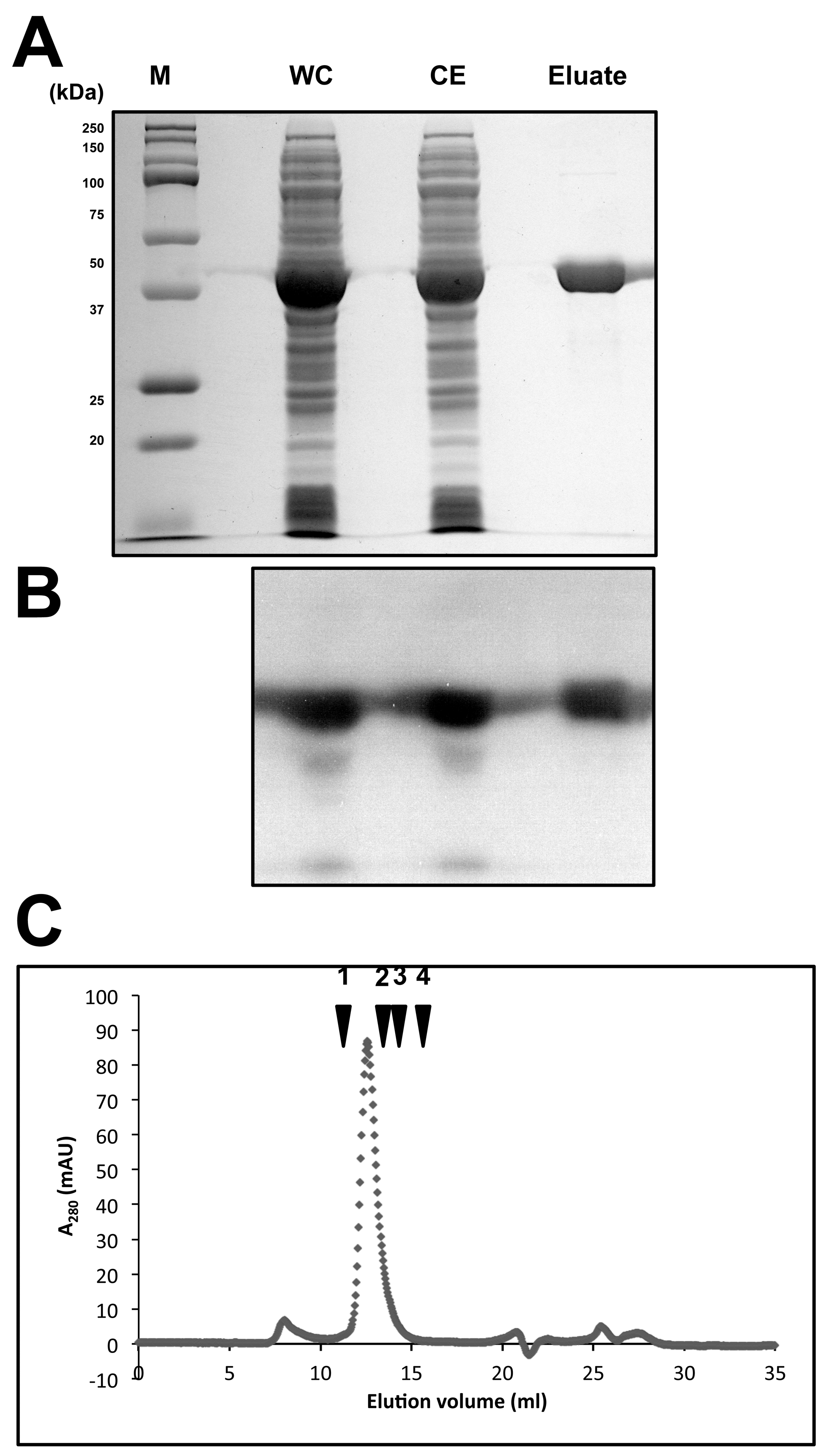

Supplement: Figure S2 — Expression and purification of hexa-histidine-tagged Tsr1. A. SDS-PAGE of rTsr1. Proteins were separated on a 10% SDS-PAGE and stained with Coomassie brilliant blue R-250. M: low range protein molecular weight marker (Bio-Rad). Molecular weights are shown on the left (kDa); WC: whole cell extract; CE: crude enzyme; Eluate, fractions eluted using 0.2 mM imidazole. B. Western blot analysis of rTsr1. Proteins in (A) were blotted, hybridized against mouse anti-His antibodies (GE Healthcare). Biotinylated goat anti-mouse IgG (Millipore Chemicon, USA) was used as secondary antibody and detection was performed with CPD-star (Roche Diagnosis) after binding with Streptavidin-AP conjugate (Roche Diagnosis). C. Gel filtration chromatography of rTsr1. Standard proteins (GE healthcare) and the purified enzyme were passed through a Superdex 200 gel filtration column (16 × 300 mm), and the relationship between molecular mass and Ve/Vo (ratio of exclusion and void volume of gel matrix) was determined. The arrowhead-labeled standard proteins are as followed: 1. Ferritin, 440 kDa; 2. BSA, 67 kDa; 3. β-lactoglobulin, 35 kDa; 4. Ribonuclease, 13.7 kDa. (TIF) [file pone.0016438.s002.tif]

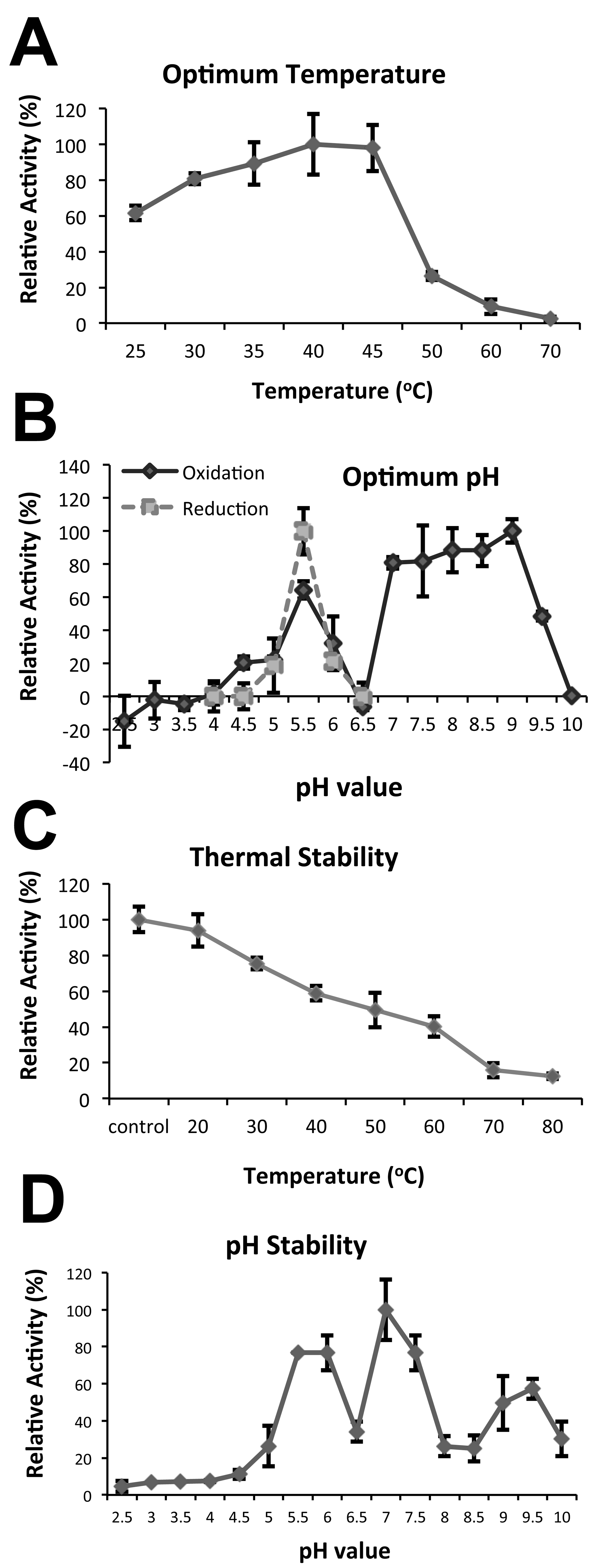

Supplement: Figure S3 — Biochemical characterization of rTsr1. A. Optimum temperature. B. Optimum pH. Oxidation and reduction reaction are marked as solid and dashed lines, respectively. C. Thermal stability. D. pH stability. Standard deviation (SD) derived from triplicates. (TIF) [file pone.0016438.s003.tif]

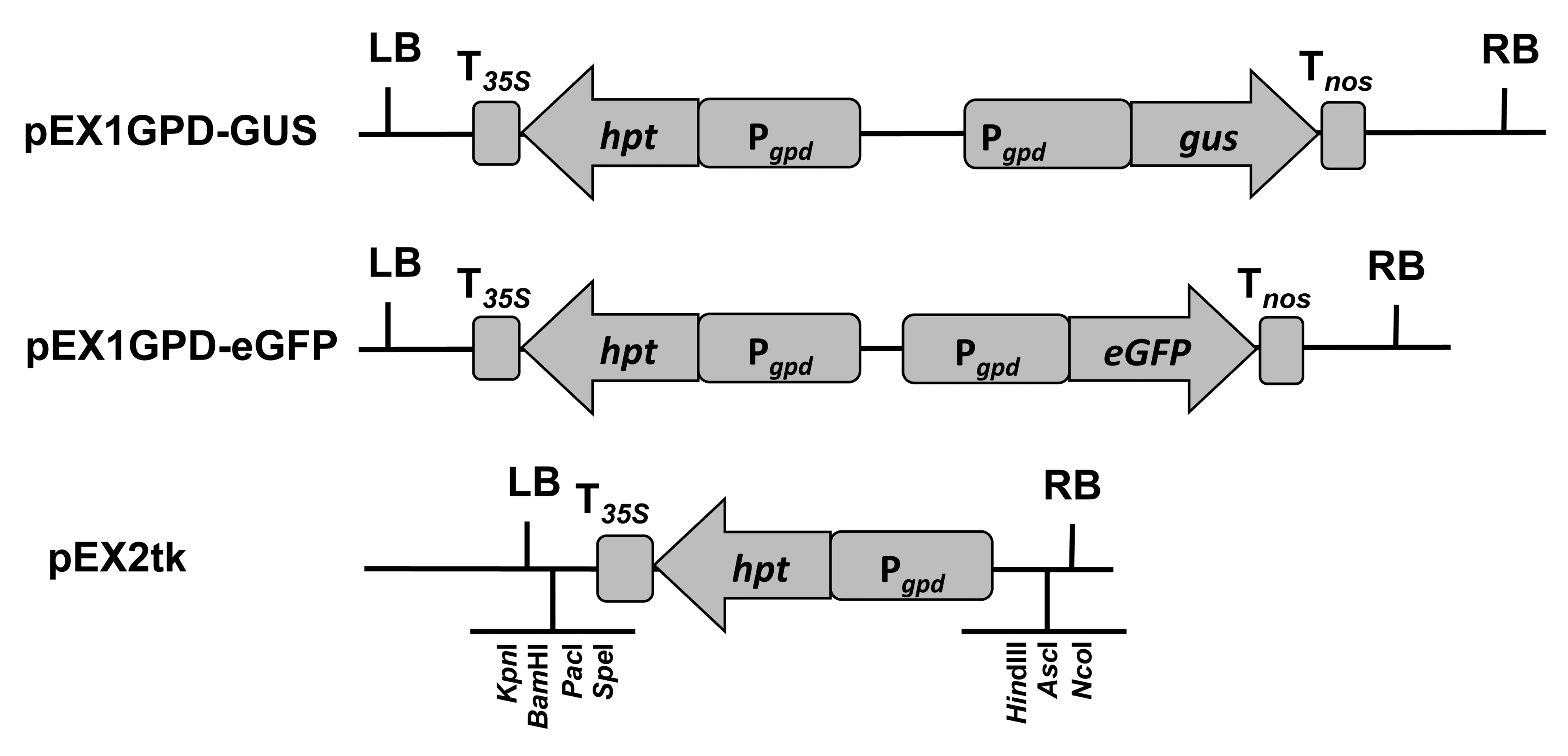

Supplement: Figure S4 — Schematic illustration of T-DNA regions of cloning vectors. A. pEX1GPD-GUS. B. pEX1GPD-eGFP. C. pEX2tk. LB: left border of T-DNA; RB: right border of T-DNA; Pgpd: promoter of gpd; hpt: Hygromycin resistance gene; gus: β-glucuronidase gene; eGFP: enhanced Green Fluorescence Protein; Tnos: terminator of nopaline synthase gene of A. tumefaciens; T35S: terminator of Cauliflower mosaic virus 35S gene; Ttef: terminator of the translational elongation factor 1 gene of Ashbya gosspii. All vectors have the same pPZP200 backbone. (TIF) [file pone.0016438.s004.tif]
