# Supplementary material for: Tartronate Semialdehyde Reductase Defines a Novel Rate-Limiting Step in Assimilation and Bioconversion of Glycerol in Ustilago maydis
Source: PLoS One. 2011 Jan 31;6(1):e16438. doi: 10.1371/journal.pone.0016438 (PMC3031564; doi:10.1371/journal.pone.0016438)
Supplement: Table S1 — Summary for purification of rTsr1. (RTF) [file pone.0016438.s005.rtf]

Table S1. Summary for purification of rTsr1
Sample	Total activity
(mU)	Total protein
(mg)	Specific activity
(mU/mg)	Yield
(%)	Purification factor	
Crude enzyme	76.2	152.3	0.5	100.0	1.0	
After Ni2+-NTA	58.2	14.2	4.1	76.5	8.2	
