# Supplementary material for: Tartronate Semialdehyde Reductase Defines a Novel Rate-Limiting Step in Assimilation and Bioconversion of Glycerol in Ustilago maydis
Source: PLoS One. 2011 Jan 31;6(1):e16438. doi: 10.1371/journal.pone.0016438 (PMC3031564; doi:10.1371/journal.pone.0016438)
Supplement: Table S2 — Effects of metal ions, chelating agent, and reducing and oxidative agents. (RTF) [file pone.0016438.s006.rtf]

Table S2. Effects of metal ions, chelating agent, and reducing and oxidative agents
Supplement	Concentration (mM)	Relative Activity (%)*	
Water	-	100.0 ± 16.7	
EDTA	5	51.6 ± 9.9	
PMSF	1	109.7 ± 2.2	
â-Mercaptoethanol	5	94.6 ± 12.2	
H2O2	5	120.4 ± 9.1	
Fe2+	1	80.6 ± 1.5	
Co2+	1	108.2 ± 8.1	
Mg2+	1	98.9 ± 6.1	
Zn2+	1	73.1 ± 12.0	
Mn2+	1	88.9 ± 13.1	
Cu2+	1	82.7 ±7.6	
Ca2+	1	90.3 ± 3.0	
Ag+	1	63.4 ± 7.6	
Pb2+	1	64.5 ± 3.0	
Li2+	1	80.6 ± 1.5	
Cs+	1	84.9 ± 1.5	
* Activity was assayed using 2 mM DL-glyceric acid as substrate and 100 M NAD+ as cofactor at 40°C.
