# Supplementary material for: Tartronate Semialdehyde Reductase Defines a Novel Rate-Limiting Step in Assimilation and Bioconversion of Glycerol in Ustilago maydis
Source: PLoS One. 2011 Jan 31;6(1):e16438. doi: 10.1371/journal.pone.0016438 (PMC3031564; doi:10.1371/journal.pone.0016438)
Supplement: Table S3 — Oligonucleotides used. (RTF) [file pone.0016438.s007.rtf]

Table S3. Oligonucleotides used

Name	Sequence	Restriction Site	
IP1	5'-ATCTGATCCAAGCTCAAGCT-3'	-	
IP2	5'-TCCCGCCTTCAGTTTAAACT-3'	-	
AD2	5'-TGWGNAGSANCASAGA-3'	-	
RBtail1	5'-GACAGGATATATTGGCGGGTAAACCT-3'	-	
Tsr1f	5'-ACGCCCAGCACGCCTCAT-3'	-	
Tsr1r	5'-CCATGGCGGGGACAAGAAA-3'	-	
Vas1f	5'-TGTCGGGCTGGACAACTGT-3'	-	
Vas1r	5'-AGTGTTCCAGGAAACGCACA-3'	-	
Ppif	5'-CTTCGACATCACCAAGAACG-3'	-	
Ppir	5'-GCGATGGTGATCTTGGACTT-3'	-	
Tsr1-Bsf	5'- TTTtcatgaCTTCTCAATCGCACCTTGGCA-3'	BspHI	
Tsr1-Br	5'- TTTggatccGAAGATGCACCGGCGTTGT-3'	BamHI	
Tsr1-Ef	5'-TTTgaattcGCTTCTCAATCGCACCTTG-3'	EcoRI	
Tsr1-Xr	5'-TTTctcgagGGAAGATGCACCGGCGTT-3'	XhoI	
Tsr1L-Nf	5'-TTTccatggTGTTTGAGCGGAAGGAGAG-3'	NcoI	
Tsr1L-r	5'-CCATGGCGGGGACAAGAAA-3'	-	
Tsr1R-f	5'-GGACCTGTTCTGTGGCGTAT-3'	-	
Ptsr1-Nr	5'-TTTccatggTTTCGAGATGAGCTTTTTCAAC-3'	NcoI	
Tsr1R-Br	5'-TTTggatccATCAGATCGGAACAGCATCC-3'	BamHI	
